# The Initial Hepatitis B Virus-Hepatocyte Genomic Integrations and Their Role in Hepatocellular Oncogenesis

**DOI:** 10.3390/ijms241914849

**Published:** 2023-10-03

**Authors:** Tomasz I. Michalak

**Affiliations:** Molecular Virology and Hepatology Research Group, Division of BioMedical Science, Faculty of Medicine, Health Science Center, Memorial University of Newfoundland, St. John’s, NL A1B 3V6, Canada; timich@mun.ca

**Keywords:** HBV, woodchuck hepatitis virus, early hepadnavirus–host DNA integration, virus-induced oxidative DNA damage, dsDNA repair, retrotransposons, oncogenesis, hepatocellular carcinoma

## Abstract

Hepatitis B virus (HBV) remains a dominant cause of hepatocellular carcinoma (HCC). Recently, it was shown that HBV and woodchuck hepatitis virus (WHV) integrate into the hepatocyte genome minutes after invasion. Retrotransposons and transposable sequences were frequent sites of the initial insertions, suggesting a mechanism for spontaneous HBV DNA dispersal throughout the hepatocyte genome. Several somatic genes were also identified as early insertional targets in infected hepatocytes and woodchuck livers. Head-to-tail joints (HTJs) dominated amongst fusions, indicating their creation by non-homologous end-joining (NHEJ). Their formation coincided with the robust oxidative damage of hepatocyte DNA. This was associated with the activation of poly(ADP-ribose) polymerase 1 (PARP1)-mediated dsDNA repair, as reflected by the augmented transcription of PARP1 and XRCC1; the PARP1 binding partner OGG1, a responder to oxidative DNA damage; and increased activity of NAD^+^, a marker of PARP1 activation, and HO1, an indicator of cell oxidative stress. The engagement of the PARP1-mediated NHEJ repair pathway explains the HTJ format of the initial merges. The findings show that HBV and WHV are immediate inducers of oxidative DNA damage and hijack dsDNA repair to integrate into the hepatocyte genome, and through this mechanism, they may initiate pro-oncogenic processes. Tracking initial integrations may uncover early markers of HCC and help to explain HBV-associated oncogenesis.

## 1. Introduction

Hepatitis B virus (HBV) and its close relative woodchuck hepatitis virus (WHV) belong to the hepadnaviral family. Both are highly oncogenic DNA viruses, and their persistent infection and integration into the host’s hepatocyte genome are the main contributors to the development of hepatocellular carcinoma (HCC) [1,2,3,4]. Primary HCC is the fifth most common neoplasm in humans, responsible for an estimated 830,000 deaths globally, and HBV infection remains the main cause of newly diagnosed HCC at an estimated rate of 56% [5,6]. In eastern North American woodchucks (*Marmota monax*) either naturally or experimentally infected with WHV, HCC develops in more than 90% of the animals with serologically evident chronic infection accompanied by protracted hepatitis [7]. This provides a highly valuable, naturally occurring animal model of progression from acute to chronic hepatitis type B (CHB), self-limited resolution of acute liver inflammation, asymptomatic occult HBV infection (OBI) and its long-term pathological consequences, and the HBV-initiated oncogenic process that often culminates in HCC [7,8]. A great advantage of this model is the stepwise progression of experimentally WHV-induced liver disease in an immunologically intact host that is inherently susceptible to a highly molecularly and pathogenically HBV-compatible virus. This infection model has been applied for basic research, preclinical evaluations of new anti-viral and anti-cancerous agents, and assessment of novel immunological approaches against CHB and HBV-associated HCC [9,10,11,12,13].

HBV infection-associated HCC typically coincides with serum HBV surface antigen (HBsAg)-positive CHB, which can be accompanied by liver cirrhosis [14]. Currently, close to 300 million people have serologically detectable chronic HBV infection, and up to a million die yearly because of HBV-caused liver diseases, including CHB, liver cirrhosis, liver failure, and HCC [15]. Nonetheless, HCC may also develop in clinically silent HBV infection (i.e., OBI), in which low levels of replicating virus persist in the absence of serum HBsAg when tested by the currently available clinical assays [3,16]. Pro-oncogenic properties of this form of silent HBV persistence have been identified in patients [3,16] and are well documented in WHV-infected woodchucks [7,17]. In this context, infection in woodchucks with doses lower than or equal to 1000 virions causes persistent trace replication of infectious WHV in the absence of hepatitis, serological (immunovirological) markers of infection, and WHV-specific B cell response while WHV-specific T cell reactivity is present [7,18,19]. This form of infection was designated as primary occult infection (POI) and found to be a cause of HCC in about 20% of animals in the context of normal liver histology and apparently unaltered hepatic biochemical function [17,18]. A similar frequency of HCC was observed in woodchucks with secondary occult infection (SOI) continuing after resolution of acute WHV hepatitis, which was accompanied by low WHV loads (i.e., typically below 100 copies/mL) in the serum and the absence of WHsAg unless ultrasensitive detection methods were applied, whereas WHV-specific antibodies and T cell responses were detectable [7,20]. The above findings indicated that HCC may develop long after the resolution of an episode of hepatitis type B and may occur even in the absence of clinical indications of liver inflammation or cirrhosis, pointing to a high carcinogenic potency of HBV.

Integration of HBV DNA into the liver genome was found to be an unwavering characteristic of HBV infection [21,22]. However, the identification of HBV–host genomic fusions and attempts to explain their role in HCC development have been essentially restricted to studies of advanced CHB with already diagnosed HCC. This led to the assumption that the formation of HBV–host DNA junctions is a rather late event that occurs randomly across the hepatocyte genome. More recent studies applying high-throughput technologies tend to indicate that some of the hepatic genes could be preferred targets of HBV insertions [23,24]. It is of interest to note that HBV and WHV also integrate into immune cell genomes and that this may contribute to the pathogenesis of lymphoproliferative disorders [8,25,26,27]. It remained unknown until recently when the first hepadnavirus–host genomic fusions are formed in infected hepatocytes, what the initial sites of HBV and WHV insertions are, which virus DNA breakpoints form merges with host sequences, and what their molecular format and mechanism of creation are. Since these very early events could be decisive in triggering liver oncogenesis, destabilizing the hepatocyte genome, and contributing to an environment supportive of pro-oncogenic perturbations that finally culminate in HCC, our laboratory investigated those issues, and the results obtained are briefly summarized in this communication.

## 2. Investigational Approaches

HBV infection experiments were carried out in hepatocyte-compatible human HepaRG and HepG2-NTCP-C4 cell lines, while WHV infection studies were performed in the woodchuck WCM260 hepatocyte-derived line and in woodchucks. The cell lines’ susceptibility to HBV or WHV infection has been established in previous studies, as reported elsewhere [28,29,30]. HepaRG cells were infected with HBV from treatment-naive patients at a multiplicity of infection (MOI) of 20 (i.e., ~2.4 × 10^7^ virus copies/~1.2 × 10^6^ cells) and analyzed for virus–host DNA fusions at 15 and 30 min (min), 1 h (h), 3 h, and 24 h post-infection (p.i.), as well as at more distant time points p.i., such as 1, 2, 3, 4, and 7 weeks [31]. In the following study, HepG2 cells overexpressing the sodium taurocholate co-transporting peptide (NTCP) receptor, identified as an HBV receptor on human hepatocytes, were exposed to recombinant HBV produced by HepG2.2.15.7 cells at an estimated MOI of 1000 (i.e., ~5 × 10^8^ virus copies/5 × 10^5^ cells), and virus integrations were examined between 15 min and 24 h p.i., as performed for HepaRG cells, and then at day 13 after infection [32]. In yet another study, woodchuck WCM260 hepatocytes were infected with wild-type WHV at an MOI of 20 (i.e., ~4 × 10^9^ virions/5 × 10^5^ cells) and investigated for WHV-host DNA fusions at 15 min, 30 min, and 1 h p.i. Cells obtained at 3 h, 24 h, and 13 d p.i. were analyzed for other markers of interest [33]. The presence of WHV–host DNA integrations was also determined in liver biopsies collected at 1 h or 3 h p.i. from woodchucks intravenously infected with a wild-type WHV with 1.1 × 10^10^ DNAase digestion-protected WHV virions [31].

**Figure 1:**
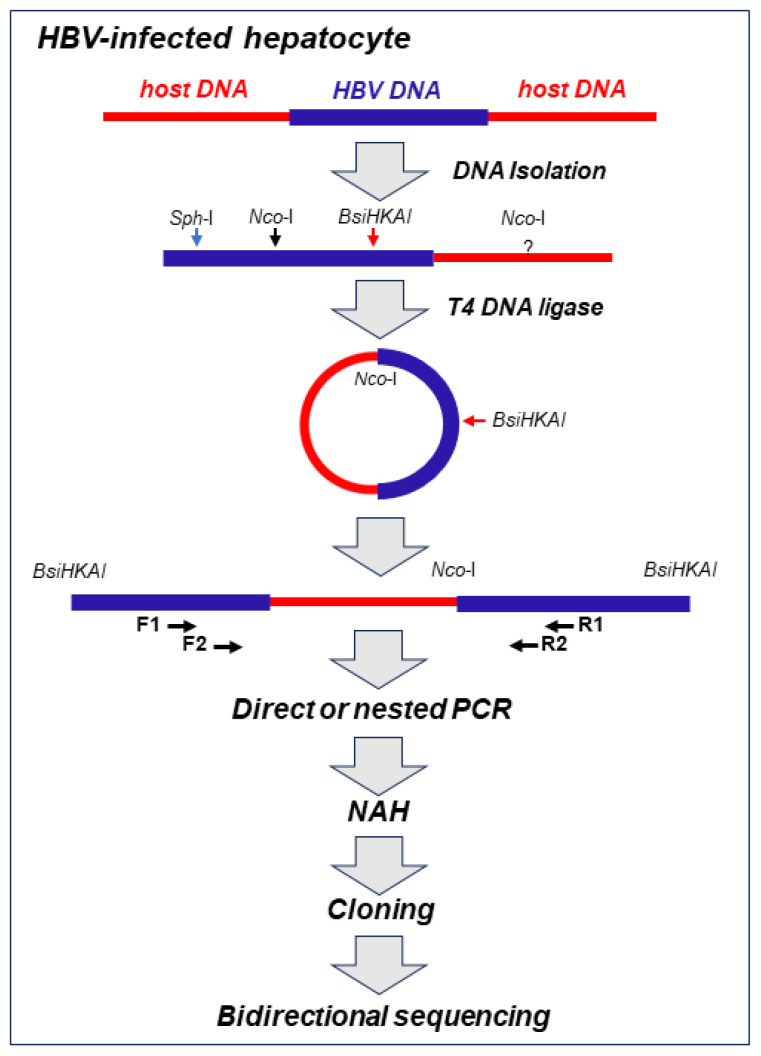
Schematic step-by-step outline of methods used for the detection of HBV integration and the identification of the sequences acting as the host’s HBV insertional sites and HBV DNA breakpoints in infected hepatocyte. Total DNA was extracted from infected cells and treated with restriction enzyme NcoI, which cut HBV DNA at the single site and the HBV-merged host’s sequence at unknown sites. The resulting hybrid DNA fragments were circularized with T4 DNA ligase and then linearized with BsiHKAI endonuclease to facilitate PCR amplification and cloning. Nested PCR was performed with forward (F1 and F2) and reverse (R1 and R2) primers. The exception was a situation in which a particular band was well identifiable after direct PCR by agarose gel electrophoresis and clearly displayed the HBV signal with nucleic acid hybridization (NAH). NAH was routinely used to verify the presence of HBV sequences and to augment the detection of the HBV–host DNA merges even when the bands carrying the merges were not apparent on the gels. This was followed by the cloning of HBV-positive amplicons and bidirectional sequencing of the clones. The aim was to sequence 20–30 clones from each band analyzed. For more information, see the text and references [19,31,32]. For identification of WHV–host DNA merges, the approach used was identical to that above, but restriction enzymes and PCR primers were specific for WHV, as reported in [19,31].

Our approach to detect virus–host genomic merges utilized HBV or WHV genome-specific amplifications by inverse polymerase chain reaction (inv-PCR). The specificity and the sensitivity of the detection of the integrated virus sequences were enhanced by nucleic acid hybridization (NAH) of the resulting amplicons with recombinant, full-length HBV or WHV sequences as probes (Figure 1) [19,31].

The employment of the NAH step increased the detection of the integrated viral sequences, which otherwise might be omitted when the gels were analyzed after electrophoresis (see Supplementary Information of reference [31]). This step also allowed the elimination of rarely occurring bands that did not contain virus-specific signals, which increased the specificity of the virus–host DNA merge detection. Only the NAH-confirmed bands carrying viral DNA were excised from the gel, and the DNA was extracted, cloned, and bidirectionally sequenced. The host and viral DNA sequences that formed junctions were identified using applicable computational software, as reported in [32,33]. A range of control experiments, including mock infections, and specificity controls ascertained the validity of the inv-PCR amplifications and the reliability of the sequencing data in each of our studies. In addition, molecular markers of HBV or WHV presence (i.e., virus genomic DNA) and replication (i.e., virus mRNA and covalently closed circular DNA, cccDNA) were assessed in the cells and liver biopsies examined. In this presentation, HBV– and WHV–host genomic fusions detected within the first hour after exposure to the virus, referred to as the initial or the earliest integrations, are the main focus, although those identified up to 24 h p.i. or later are occasionally discussed to provide a wider picture of our findings.

## 3. Virus–Host Genomic Fusions Emerging in the First Hour after Infection

**Figure 2:**
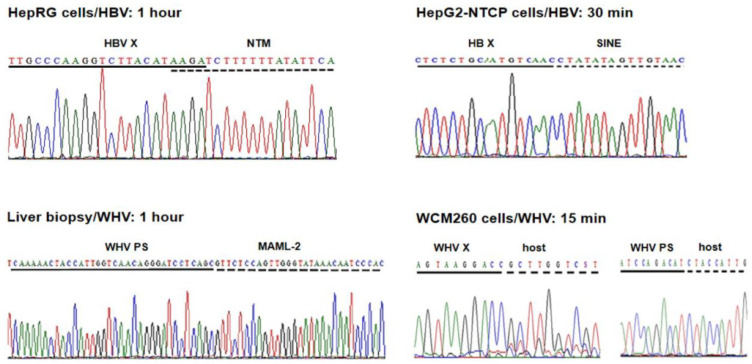
Examples of the earliest HBV and WHV DNA fusions with human or woodchuck genomic sequences detected between 15 min and 1 h after exposure to virus in different infection systems investigated in our studies. With the exception of the overlapping homologous junction (OHJ) created by the HBV X gene (HBV X) and human neurotrimin (NTM) gene displayed on the left side of the top panel, all other virus–host genomic fusions shown have the format of the head-to-tail junction (HTJ). In the bottom left panel, the virus–host DNA merge was formed by the preS sequence of WHV S (envelope) gene (WHV PS) and it was detected in a liver biopsy obtained from a woodchuck one hour after injection with WHV, as described in the text. Other abbreviations: SINE, short-interspersed nuclear element (retrotransposon); MAML-2, mastermind-like 2; WHV X, WHV X gene; host, unidentified woodchuck genomic sequence.

**Figure 3:**
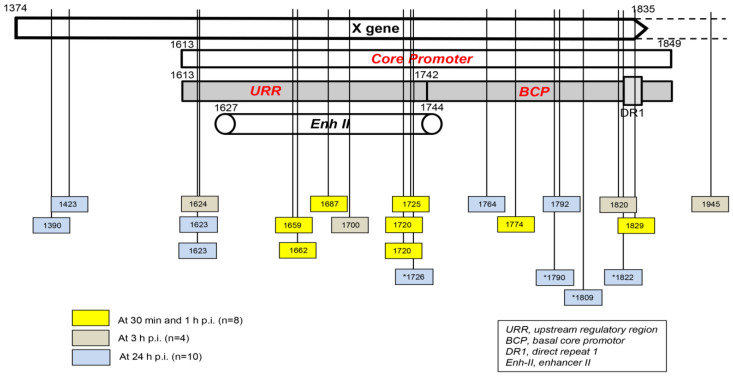
Schematic presentation of the HBV X gene break points forming fusions with the human genomic sequences that were detected in hepatocyte-compatible HepRG and HepG2-NRCP-C4 cells within the first 24 h after exposure to HBV. Yellow squares show breakpoints that formed junctions detected at 30 min and 1 h after exposure to virus, red squares show those identified at 3 h post-infection, and blue squares show those detected at 24 h post-infection. Blue squares with stars show breakpoints reported at 24 h after infection in reference [36]. Numbers mark nucleotide positions according to the HBV DNA GenBank X79185 sequence. Abbreviations: URR, upstream regulatory region of the HBV core promoter; BCP, basal core promoter; DR1, direct repeat region; Enh II, HBV enhancer II.

Five HBV DNA insertions into five distinct genes were identified at 1 h p.i. in HepaRG cells exposed to authentic, patient-derived HBV [31]. Four of them were detected by single hits (i.e., in a single clone), while one was identified by multiple hits (i.e., in eight clones). This last site was in the human neurotrimin (NTM) gene located on chromosome 11q25. This gene encodes a neural adhesion molecule, and its expression was also found in stem cells and hepatic stellate cells [34]. The fusion was created by a four base-pair overlapping homologous junction (OHJ) with the HBV sequence located in the upstream regulatory region (UPR) of the HBV X (HBx) gene that carries HBV enhancer II (Enh-II) (Figure 2; see Figure 3 for more information). Due to the fusion with the enhancer, this HBV insertion may potentially modify hepatocyte function and influence pro-oncogenic processes. Notably, the OHJ format of the HBx–NTM fusion was the only one of this type detected in our studies within 1 h of infection. All remaining HBV– or WHV–host genomic merges demonstrated the format of head-to-tail junctions (HTJs) (Figure 2). In the same study, liver biopsies acquired at 1 h or 3 h p.i. from woodchucks intravenously injected with WHV were also examined. Overall, seven unique WHV DNA insertions into different host genes were detected at 1 h p.i. and three more were detected at 3 h p.i. [31].

Together, the time of the first hepadnavirus DNA integration into the hepatocellular genome was identical in human HepaRG cells exposed to HBV and in woodchucks infected with WHV. Regarding the replication of HBV and WHV, it was detected from 1 h p.i. in both infection systems [31].

HepG2-NTCP-C4 cells exposed to recombinant HBV at an MOI of 1000 provided a more effective system for the identification of virus–host integrations, as judged by the twice shorter time of the appearance of the first virus–host genomic fusions [32]. The earliest HBV insertions into two distinct genes were identified at 30 min p.i., and one more fusion with another gene was detected one hour after exposure to the virus. One of the sites was a merger with a retrotransposon called a short-interspersed nuclear element (SINE) (Figure 2). The second was with the neuroblastoma breakpoint family member 1 (NBPF-1), a pseudogene that functions as a tumor suppressor for neuroblastomas [35]. In addition, HBV insertion into a retrotransposon termed the mammalian apparent retrotransposon long terminal repetitive (THE-1B-LTR) element was detected at 1 h p.i. (see below). There were no HBV DNA integration signals found in HepG2-NTCP-C4 cells collected at 15 min p.i. or cells subjected to control mock infections, but there were several more HBV DNA insertions identified in the cells harvested beyond 1 h p.i., as reported in [32]. HBV replication in HepG2-NTCP-C4 cells became evident from 1 h p.i., indicating that the detection of virus integration preceded the appearance of virus nucleic acid replicative intermediates.

The earliest WHV–host integrations were also investigated in woodchuck WCM260 hepatocytes exposed to wild-type WHV [33]. In this model, four different host genes were identified as the initial integration sites at 15 min p.i. (Figure 2). In addition, four other fusions were detected at 30 min p.i., and three more were detected at 1 h p.i. This showed that hepadnaviral insertions occur, in fact, immediately after the hepatocytes first contact the infectious hepadnavirus and confirmed that this takes place before the initiation of virus replication, as some preceding studies have also indicated [32]. This remarkably short time between the first contact with the hepadnavirus and its DNA insertion into the hepatocyte genome became less startling when the kinetics of virus-triggered cell DNA damage and the kinetics of the damage repair were recognized (see below).

## 4. Retrotransposons and Transposable Elements Are Frequent Targets of the Early HBV Integration

The clonal sequencing of the early integration sites showed that, in addition to several host somatic genes creating HBV–host fusions, mobile genetic elements containing repetitive non-coding genomic sequences, such as retrotransposon and transposon elements, and genes with translocation potential were frequent virus integration targets [31,32]. In our study on HBV-infected HepaRG cells, HBV DNA insertions within or near the long-interspersed element-1 (LINE1 or L1) and LINE2 (or L2) sequences were identified in the first 24 h p.i. [31]. HBV fusions with LINE1 were also detected at 24 h p.i. in Huh7-NTCP hepatocyte-like cells investigated by others [36]. HBV also formed a fusion with the gene encoding fibronectin leucine-rich transmembrane protein (FLTR2), which, at its C-terminus, was joined with the LINE2 sequence. These joints were found at 3 h and 24 h p.i. as well as on day 3 p.i., and all three targeted the same locus on chromosome 14. Since over twenty percent of the human genome is comprised of the LINE sequence, the finding of the HBV–LINE fusions is not surprising [37]. In this regard, LINE1 constitutes as much as 18% of the genome, while LINE2 accounts for about 3% of the genome [37]. LINE1 displays endonuclease and reverse transcriptase activities and transposes via target-primed retrotransposition [37]. These properties underline LINE1’s pro-oncogenic potential. It has been reported that transcripts of HBV X gene–LINE1 chimeras have tumor-fostering characteristics and may adversely affect the survival of patients with HBV-associated HCC [38]. HBV integrations into the LINE sequences have been found in large numbers in tumorous and non-tumorous liver tissues from patients with HBV-associated HCC [39]. Notably, while fusions of the HBV X gene with LINEs occurred at significantly higher numbers in HCC tissue, the merges with HBV S gene sequences were more frequent in non-tumor tissue. It also is of interest to mention that beyond 24 h p.i., HBV DNA fusions with a retrotransposable element, annotated as human satellite II (HSAT-II) DNA, were identified in HepaRG cells [31]. This merge was particularly well represented on day 14 p.i. considering the number of clones in which it was identified. The integration of HBV with the HSAT-II sequence has not been reported previously, but integration with the similar HSAT-III was identified in human hepatoma cells and patient-derived HCC tissue [31,40].

In HepG2-NTCP-C4 cells infected with HBV, one of the two earliest insertional sites detected at 30 min p.i. was a junction with the retrotransposon SINE, as already mentioned. The HBV–SINE joint was found in 85% of the clones carrying the initial HBV–host DNA integrations in this infection system. This retrotransposon belongs to the non-long terminal repetitive (non-LTR) category. It is abundantly represented in the human genome and implicated in oncogenic transformation [41]. One hour after exposure to HBV, HBV fusion with THE-1B-LTR was detected. This element is included in the mammalian apparent LTR retrotransposon (MaLR) family and is involved in the pathogenesis of non-Hodgkin’s lymphoma [42]. THE-1B-LTR was joined with the HBV Enh-II sequence, and therefore, this hybrid might have a pathogenic relevance by modulating hepatocyte functions and possibly advancing the development of HCC. On day 13 p.i., other HBV direct integrations or indirect merges with retrotransposons or transposable elements were detected in HepG2-NTCP-C4 cells. One of them was HBV merging with hobo activator-18 *Salmo salar* long terminal repeat (hAT-18-SsA), which transposes across the genome via DNA-DNA fusions [43]. hAT-18-Ssa was joined by a non-coding sequence of chromosome-2, and that, in turn, was fused with the medium reiterated frequency repeat 5B (MER-5B), which also acts as a transposon. Thus, this trimera was formed by the HBV sequence and two transposons. It is known that MER-5B controls the expression of the gene encoding alpha fetoprotein (AFP), which occurs at high levels in fetal liver and HCC [44]. This may suggest a connection between HBV integration and increased AFP synthesis, which might be worthy of further investigation. Furthermore, HBV DNA–LINE2 fusion was identified at the same time point after infection. Overall, among the HBV–host genomic junctions identified in HepG2-NTCP-C4 cells, 33% of all merges were with transposable elements, and 49% of the clones carrying this type of merges belonged to the category of initial or very early HBV integrations. When the results from the HepRG and HepG2-NTCP-C4 cell lines were compared with the data from HepG2-NTCP and Huh7-NTCP investigated by others between 24 h to 7 days p.i. [36], HBV merges with the same or comparable transposable elements were identified. These common elements included LINE1, SINE, THE-1B-related THE-Int, and MER-5B-related MER52D/41A/90A/4E1/4A [32]. Therefore, there was relatively good compatibility between the findings obtained from different HBV infection models.

Our study documented that HBV insertions into hepatocyte repetitive non-coding sequences, such as retrotransposons and transposons, are frequent among initial integration sites. They became detectable within 30 min of virus exposure and before molecular evidence of virus replication was apparent. The engagement of these mobile elements may suggest a mechanism by which HBV DNA could be dispersed across the hepatocyte genome soon after its entry into the hepatocyte. The transposition of the fused HBV-mobile elements, in particular merges with virus regulatory sequences (see below), may expand the spread of virus genomic material throughout chromosomes as well as perturb the organization and transcriptional activity of the hepatocellular genes. In consequence, hepatocyte function could be altered from the beginning of infection, predisposing the cell to pro-oncogenic changes during the cell’s lifetime, which may prompt HCC development in some cases. This is consistent with the observed increase in HBV integrations within transposable elements in the livers of patients with HBV-associated HCC [38,39,45,46].

## 5. Hepadnavirus DNA Breaking Points Yielding Early Fusions with Hepatocyte Genome

The localization of HBV and WHV DNA breakpoints creating junctions with host sequences in the first hours after exposure to the virus was limited to the X gene sequence owing to the design of the primers applied for inv-PCR amplifications. Undeniably, DNA breaks also occur in other parts of HBV and WHV genomes, and they can create merges in the initial stages of infection. This was already exemplified when primers for the WHV preS genomic region were used to identify WHV DNA insertions and breakpoints in the preS and P gene sequences in liver biopsies acquired from woodchucks at 1 and 3 h p.i. (see Figure 2) [31].

Taking into consideration the data from HepaRG and HepG2-NTCP-C4 cells obtained up to one hour after exposure to the virus, there were eight HBV breakpoints found in total [31,32]. Six of them occurred within the X gene fragment overlapping the Enh-II sequence, and the remaining two occurred in the base core region (BCR) of the core promoter (Figure 3). There were also 10 other breakpoints detected at 3 h and 24 h after infection. Only one of them, detected at 3 h p.i., was located within Enh-II; three were detected in the upstream regulatory region (URR) of the core promoter just before the Enh-II sequence, three more were detected within the BCR, two were detected at the beginning of the X gene sequence, and one was detected outside the X gene at the beginning of the core gene (Figure 3). By analyzing the data reported by another group, which were collected at 24 h after the infection of Huh7-NTCP cells with HBV [36], we found that one of the four HBV breakpoints was within the Enh-II sequence, and the remaining three were within the BCR. Overall, the results gave the impression that the earliest integration sites identified at 30 min and 1 h p.i. were created mainly by the breakpoints emerging in the HBV Enh-II sequence (Figure 3). By contrast, the BCP appeared to be the most prone to DNA breakages one hour after exposure to the virus, i.e., at 3 h and 24 h p.i. This might be an interesting observation since both Enh-II and the BCR are important regulatory elements relevant to HBV replication, and they may also modulate the expression of the merged host sequences. The analysis of more breakpoints within these elements and the recognition of the transcriptional activity and functional consequences of the resulting chimeras are required before this possibility becomes less elusive. WHV DNA breaks forming junctions with woodchuck genomes in liver biopsies collected in the first 3 h p.i. were also predominantly located in the WHx gene fragment overlapping the WHV BCP sequence, and five different breakpoints were found at this location [31]. In WCM260 hepatocytes exposed to WHV, virus integrations were discovered as early as 15 min p.i., and all WHV breakpoints found at this time point were located within the virus BCP sequence (Chauhan and Michalak, data unpublished).

## 6. Molecular Format of the Earliest Hepadnavirus–Host Genomic Junctions

The great majority of the HBV– and WHV–host genomic fusions detected in the first hour p.i. were merges of the HTJ type. By contrast, those with the OHJ format were very rare (Figure 2) [31,32,33]. Thus, among 8 HBV and 19 WHV insertional sites identified during this time period, only one (3.7%) was of the OHJ type, and it was created by HBV and the NTM gene sequence, as mentioned above [31]. The same situation was seen when the host sites of HBV or WHV insertions were enumerated between one and 24 h p.i. Thus, a further nine HBV and three WHV sites were identified. Among them, again, only one merge displayed the OHJ format, implying that it was created by micro-homology overlapping joining (MHOJ). This particular fusion was formed by HBV and the runt-related transcription factor 1 (RunX1) sequence at 24 h p.i. in HepG2-NTCP-C4 cells [32]. The HTJ formats of four other HBV–host fusions were identified in Huh7-NTCP cells exposed to recombinant HBV and investigated at 24 h p.i. by another group [36]. Taken together, the results showed that HBV and WHV, in the earliest stages of infection, are joined with hepatocyte genomic sequences almost entirely via HTJs. The creation of such junctions reflects the involvement of the non-homologous end-joining (NHEJ) mechanism [47,48]. Directed by this finding and by the fact that NHEJ is involved in the repair of cell dsDNA breaks, the presence of which is a prerequisite for HBV DNA integration [49], we wanted to identify a molecular thread connecting these events.

## 7. Mechanism of Formation of the Initial Hepadnavirus–Host Genomic Merges

**Figure 4:**
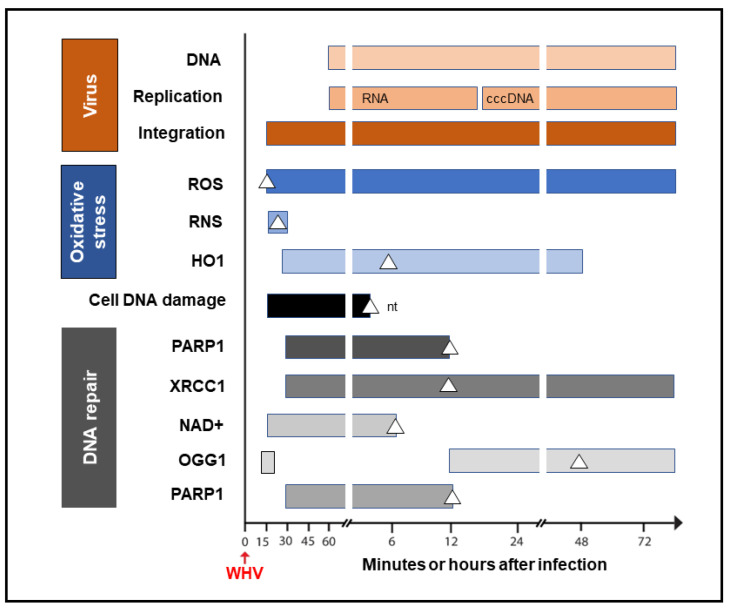
Graphic presentation of the earliest detections and changes over time in the presence of virus genome (DNA), its replication (mRNA and cccDNA) and integration into the hepatocellular genome, indicators of hepatocyte oxidative stress and oxidative DNA damage, as well as markers of the activity of components of the DNA repair pathway during the first 72 h after exposure to HBV or WHV. The profiles represent cumulative data from HBV and WHV infections in human and woodchuck hepatocyte-compatible cells and in woodchucks infected with WHV. White triangles mark the time at which peak expression or activity was found. Whites square in the OGG1 line represent a 5.6-fold increase in the gene expression over uninfected control cells, which did not achieve a statistically significant difference. For more details, see reference [33]. Abbreviations: cccDNA, virus covalently closed circular DNA; ROS, reactive oxygen species; RNS, reactive nitrogen species; HO1, heme oxygenase-1; PARP1, poly(ADP-ribose) polymerase 1; XRCC1, X-ray repair cross-complementing protein 1; NAD^+^, nicotinamide adenine dinucleotide; OGG1, 8-oxyguanidine DNA glucose 1, and nt, not tested beyond this time point.

It was shown that HBV could induce oxidative stress by triggering the production of reactive oxygen species (ROS) and reactive nitrogen species (RNS) that cause cell DNA oxidation and oxidation-induced double-stranded (ds) DNA breakages [50]. To assess whether oxidative DNA damage contributes to the formation of the initial hepadnavirus–host DNA junctions, woodchuck WCM260 hepatocytes in which WHV–host HTJs were formed from 15 min p.i. were investigated from this time point onward. For details on the kinetics of the individual markers of oxidative stress, cell DNA damage, and DNA repair, please see Figures 2–5 included in reference [33]. Thus, we found a strong and prolonged induction of ROS but only very transient production of inducible nitric oxide (iNOS), which both became detectable from 15 min after exposure to the virus (Figure 4). Notably, while ROS reactivity remained highly elevated between 15 min and 6 h after infection, the activity of iNOS increased only briefly between 15 and 30 min p.i. (see Figure 2 in reference [33]). This coincided with microscopically evident cellular DNA damage, which significantly increased between 15 min and 1 h p.i., as determined by single-cell alkaline comet assay and the nuclear tail length measurements (see Figure 2 in reference [33]). To determine whether the repair of the DNA breakages due to virus-induced oxidative stress was responsible for the formation of the initial virus–host fusions, the time kinetics of the transcription of poly(ADP-ribose) polymerase 1 (PARP1), which recognizes dsDNA breaks and facilitates their repair by the alternative NHEJ pathway, and X-ray repair cross-complementing protein 1 (XRCC1), which is a binding partner of PARP1 in this process, were quantified (see Figure 4 in reference [33]). This was supplemented by the quantification of nicotinamide adenine dinucleotide (NAD^+^) activity, an indicator of PARP1 activation; the transcription of heme oxygenase-1 (HO1), a marker of pro-oxidative cell stress; the transcription of 8-oxyguanidine DNA glucose 1 (OGG1), an indicator of cell response to oxidative DNA damage; and the quantification of the PARP1 cleavage activity (see Figures 4 and 5 shown in reference [33]). The data revealed the synchronized induction of PARP1 and XRCC1 transcription accompanied by augmented reactivity of NAD^+^ and HO1, which all were initiated at 15 to 30 min p.i. (Figure 4).

In addition, a transient 5.6-fold increase over uninfected control cells in the OGG1 gene expression (although not statistically significant) was detected between 15 and 30 min p.i. and then from 12 h p.i. onward [33]. PARP1 cleavage became significantly augmented between 6 and 12 h p.i., but a twofold increase, although not statistically significant, was identified 1 h after infection. These quantitative measurements indicate that hepadnavirus is a strong and essentially immediate inducer of hepatocyte oxidative stress and associated DNA damage and that the PARP1/XRRC1-initiated dsDNA NHEJ repair is involved in the creation of the earliest virus–host fusions. A progressive increase in PARP1 transcription up to 6 h p.i. subsided after 12 h p.i. to the level detectable in control cells (Figure 4). This sharp decline was accompanied by an increase in the cleavage of PARP1 protein (see Figure 5B presented in reference [33]). Jointly, our data show that PARP1-facilitated dsDNA repair is engaged in the initial stages of infection, and the NHEJ mechanism determines the HTJ format of the earliest hepadnavirus–host genomic merges. In stages beyond a few hours after infection, the virus–host junctions tend to display the OHJ format more often, implying their creation by the MHOJ repair mechanism.

## 8. Conclusions and Future Directions

The propensity of HBV to integrate into the human hepatocyte genome has been recognized from the commencement of studies investigating this virus, but the exact time at which this happens, the molecular format of the earliest virus–host genomic fusions, and the mechanism of their formation remained unrecognized until now. Because HBV DNA integration into the hepatocellular genome is considered to be the main contributor to this virus’s oncogenicity, this prompted our interest in the elucidation of the time kinetics of creation of the earliest HBV– and WHV–host DNA merges, the identification of the host sequences that function as the sites of virus initial insertions, and the mechanism of creation of these earliest virus–host junctions. The data obtained showed that the first HBV insertions became detectable within 30 min of infection with HBV in human HepG2 cells overexpressing NTCP [32] and at 1 h after exposure to HBV when human HepaRG cells and liver biopsies from woodchucks infected with WHV were examined [31]. Human non-coding DNA elements, including retrotransposons and transposons, constituted almost half of the early HBV insertional sites. In addition, several somatic genes, including those coding factors and enzymes implicated in cell growth and cancer development, were identified at the very early time points post-infection. A similar study of woodchuck hepatocytes infected de novo with WHV showed virus DNA insertions from 15 min onward after exposure to the virus. This further revised our perception about the time required for the formation of the first virus–host genomic fusions and solidified the data indicating that hepadnavirus integration can precede the initiation of its replication. This prompted our studies on the molecular mechanism of the formation of the initial virus–host DNA merges. Our findings showed that the initial fusions had almost exclusively the head-to-tail format, which indicates that their formation involves the NHEJ pathway. We elected to examine the alternative route for NHEJ dsDNA repair that is mediated by PARP1, which is known to operate in response to oxidative dsDNA damage in mammalian cells and is prone to joining errors, potentially leading to cancer [48,51]. The examination of intracellular levels of ROS, cellular DNA damage, and the transcription of genes involved in the response to oxidative DNA damage unveiled that their statistically significant increases occurred 15 to 30 min after infection, and hence, they closely coincided with the formation of the first hepadnavirus–host fusions [33]. These results collectively showed that hepadnavirus is a potent and instantaneous trigger of oxidative DNA damage in hepatocytes and swiftly activates dsDNA repair machinery. This machinery facilitates the incorporation of viral DNA sequences at breakpoints into host DNA and determines the prevailing head-to-tail format of the initial DNA junctions. Interestingly, HBV and WHV regulatory elements in their X gene sequences appear to be most prone to breakage and the formation of the earliest virus–host DNA fusions. This could modify the expression of individual genes and compromise the overall stability of the hepatocyte genome, thereby creating a microenvironment supportive of the oncogenic process that, with time, may culminate in HCC in some cases. It is feasible that a similar pro-oncogenic process can be initiated by HBV at the extrahepatic locations in which virus–host genomic merges are formed [8,19,27].

Several aspects recognized or suggested by the studies summarized in this communication require further investigation. Among them, complete mapping of the integration sites and the DNA breakpoints within the HBV and WHV genomes forming initial junctions with host sequences would help to determine whether other hot spots, similar to the X gene/core promoter sequence, exist. This would confirm or indicate a need to modify the scope and the focus of further investigations. In this regard, the determination of the ability of different HBV genotypes to form early virus–host fusions, particularly genotypes that coincide with HCC development more frequently than others, might be interesting. We noticed two profiles of initial HBV integration in our earliest study [31]. Thus, while the HBV inoculum of the A genotype yielded within 1 h of infection merged with several genes identifiable in singular clones, HBV of the C genotype produced fusions with two sites, which were robustly represented in multiple clones within the same time period. Since the experimental and analytical conditions were identical, this variation might be linked to the virus alone. This preliminary observation requires further study to become conclusive. Another issue awaiting explanation is how the HBV and WHV are sensed by hepatocytes, enabling the triggering of swift oxidative DNA damage. Thus, the involvement of a viral protein, a signal sent by virus-induced cell protein conformational change, an alteration or unmasking of a particular viral DNA structure immediately after virus entry, or another unknown mechanism must be established in further studies. Furthermore, the finding of numerous and instantaneous HBV and WHV insertions into host somatic genes and noncoding sequences raises important issues regarding their long-term stability and possible functional and pathogenic consequences. Essentially there are no data in this regard except some indirectly applicable information derived from the analysis of HBV–host junctions in nontumorous and tumorous liver tissues in advanced CHB. One such observation is that basically the same HBV X gene/core promotor region is prone to breakpoints both immediately after virus entry into the hepatocyte (see Figure 3) and in HBV infection persisting for years in the liver [31,52,53]. As proposed for chronically infected livers, the initial or very early HBV insertions into the hepatocyte genome may predispose to the clonal expansion of the cells carrying certain chimeric sequences [53,54]. Considering the above, although it is difficult to imagine the survival of the initially infected hepatocyte for years due to its normally limited lifespan (unless immortalized), it is more acceptable to consider that daughter cells created throughout clonal expansion carry the same or partially the same (due to subsequent insertions at new sites) HBV integration profile as the primarily infected hepatocytes. The overlapping ranges of the host sequences serving as HBV initial and very late (i.e., during CHB) insertional sites might be interpreted in support of this possibility. Regarding the delineation of potential functional and pathogenic/oncogenic consequences of the initial and very early virus–host chimeras, there is progress in exploring approaches allowing the examination of such issues. This is exemplified by the identification of transcriptional activity of the HBx–LINE1 chimeras in patients with HBV-associated HCC and by showing tumor-promoting properties of the transcripts in vitro [38,55].

## Data Availability

Not applicable.
